# Stem cells treatment in the ocular surface regeneration

**Published:** 2017

**Authors:** Cristina Nicula, Izabela Szabo, Ozana Ivan

**Affiliations:** * “Iuliu Hatieganu” University of Medicine and Pharmacy Cluj-Napoca, Romania; ** Eye Clinic Cluj-Napoca, County Emergency Hospital Cluj-Napoca, Romania

**Keywords:** stem cells, graft, corneal surface

## Abstract

The article presents the modern treatment with stem cells in the reconstruction of ocular surface. The turnover of the stem cells, the location in the limbus areas, the importance of limbal stem cells presence, the clinical appearance of stem cell deficiency, investigations method for this illness and the management of stem cell deficiency (artificial lacrimal tear drops, mini scleral contact lenses and the surgical treatment with allografts and autografts of stem cells) were taken into account.

Many tissues in the adult organism undergo rapid and continuous cell turnover. These tissues (simple and stratified epithelium) must repopulate and simultaneously maintain the integrity of the tissue. Stem cells have a high capacity for self-renewal extending through adult life [**1**]. They represent 0,5% or less to 10% of the total cell population [**2**,**3**].

The cornea forms part of the ocular surface of the eye. It is formed of epithelium, Bowmann layer, stroma, Descemet membrane, and endothelium. The epithelium forms 10% of the corneal thickness (basal cells, wing cells, squamous cells), its role being to absorb nutrients and oxygen and to protect the eye. Bowmann layer is an acellular zone of the anterior stroma located just beneath the basement membrane. The stroma forms 90% of the corneal thickness, it is avascular and contains glycosaminoglycans and proteoglycans, water, collagens interspersed with keratocytes or fibroblasts. Descemet membrane is the basement membrane of the endothelium and its role is to pump excess water out of the stroma, maintaining the corneal transparency. 

The epithelium has a constant process of cell renewal and regeneration. Cells in the upper most layer of the corneal epithelium are continuously desquamated from the surface and must be replaced by cell proliferation [**4**-**6**]. Only cells that are in contact with the basement membrane have the ability of mitotic cell division. Cells that are displaced into the suprabasal layer become postmitotic and lose their capability for cell division [**4**,**5**].

There are two controversial theories regarding the proliferation of basal cells: the first theory states that the origin of corneal epithelium cell proliferation is derived from the adjacent conjunctiva by conjunctival transdifferentiations [**4**-**6**], while the second says that the origin of corneal epithelium cell proliferation depends on corneal stem cells in the limbal basal epithelium [**4**-**6**].

The location of the corneal epithelial stem cells in the limbus is not certain. In 1971, Davanger and Evensen [**7**] affirmed that pigmented cells in the limbus migrated centripetally towards the central cornea, so stem cells are localized in the basal cell layer of the limbus. Schermer et al. [**14**] showed that corneal epithelium originates from the limbus. Limbal basal epithelium contains the stem cells of the corneal epithelium. Catsarelis et al. [**7**] said that only limbal basal cells retain thymidine label for long periods, with long cell cycle time. Ebato and Lindberg [**8**,**9**] showed that limbal basal cells have a higher proliferative potential in culture than central corneal epithelial cells. Chen [**10**] demonstrated that the surgical removal of the limbal region results in healing with noncorneal epithelium. Kenyon [**11**] showed that limbal transplants results in the regeneration of cornea-like epithelium. Catsarelis/ Zietske [**12**] suggested that the limbal basal cells answer to central cornea wounds by undergoing cell division (as expected from stem cells).

Little is known about the mechanism that helps maintain and perpetuate the stem cells in the limbus. The extrinsic and intrinsic properties have to be taken into consideration. The extrinsic properties are characteristics of the environment surrounding the stem cell. The maintenance of “stemness” by extrinsic properties is explained by a model proposed by Scofield (1983) [**13**], which showed that stem cells exist in an optimal “niche” that promotes the maintenance of stem cells in an undifferentiated condition. After the division, only 1 cell (daughter) can reenter the niche, the other one differentiates and becomes transient amplifying cells (TA). Following the division of the stem cell, the daughter cells can either reenter the stem cell niche or enter a less niche that allows the cell to remain undifferentiated and retain a stem-like characteristic like following division. These cells can enter the differentiation pathways or remain in an undifferentiated stem-like state. Cells leaving the “niche” have the capability to divide.

If the limbus contains a stem cell niche, then the structure is different from the central cornea.

The limbal zones formed from blood vessels (nutrition of the limbus, interaction with blood cytokine [**15**], characteristic of stem cells [**16**]) and anchoring fibrils, which extend from the basement membrane and intersect with other anchoring fibrils extending through the stromal pegs, which form a niche promoting the adherence of the limbal basal cells, protecting them from physical injury. 

The limbal basement membrane is composed of type IV collagen [**16**]. There is an antibody AE-27 [**16**], which bounds central corneal basement membrane strongly, weakly conjunctiva, and limbus heterogeneously. It can express K3 (differentiation marker), building AE-27 at high level. This is not seen at the corneal level.

The intrinsic properties of the limbal stem cells are faster proliferation in culture [**16**], growth factors and calcium ions affect the cell types differently [**16**], limbal epithelium is more resistant in tumor promoters [**16**], transplant of limbal epithelial cells resulted in growth of a limbus-like epithelium [**16**], they contain high levels of several proteins as metabolic enzymes (alpha enolase), cytochrome oxidase, Na/ K-ATP-ase, carbonic anhydrase [**16**], limbal cells may be more metabolic and intermediate filaments, like vimentin, Keratin 19 [**16**] may be responsible for the anchorage of the stem cell into a certain environment. 

## The importance of corneal stem cells for the regeneration of the corneal epithelium

The location of the stem cells in limbal basal epithelium is dependent on the integrity of the limbus.

The arguments regarding the importance of corneal stem cells for the regeneration of the corneal epithelium are the fact that the original corneal phenotype cannot be maintained in the absence of stem cells from the limbus and that the original phenotype of corneal epithelium can be reconstituted by surgical transplantation of limbal stem cells.

In cases of wound healing in the absence of corneal stem cells, Tseng et al. [**5**,**6**] showed that in the presence of corneal stem cells within an uninjured limbal epithelium, corneal epithelium regenerates despite repeated small central wounds, even if the total corneal epithelium is removed. In the partial absence of limbal corneal stem cells (limbal deficiency), the remaining stem, and transient amplifying cells, they maintain the corneal epithelium under physiological circumstances and regenerate the central corneal epithelium. Chen et al. [**6**] found that the removal of the TA cells in eyes with partial limbal deficiency leads to a delayed wound healing, vascularisation, expression of a conjunctival phenotype. TA cells are important in the maintenance and regeneration of the corneal epithelium, even in the absence of stem cells. Remaining stem cells could not regenerate enough TA cells to reconstitute the corneal epithelium. 

## Transplantation of corneal stem cells

In case of transplantation of corneal stem cells, Kenyon and Tseng [**4**-**6**] discovered that the original corneal phenotype could be reconstituted by the transplantation of healthy corneal stem cells. Based on the concept that a simultaneous loss of corneal stem and TA cells causes the alteration of the corneal phenotype, Tsai et al. [**4**-**6**] showed that in the case of a simultaneous removal of limbal and corneal epithelium, conjunctivalization and neovascularization will appear.

## Regulation of corneal stem and transient amplifying cells

Conversion of stem cells in TA cells is supported by serum factors, such as retinoic acid. The amplification of TA cells is promoted by epidermal growth factor (EGF), acidic and basic fibroblast growth factor 1 (a; b FGF), nerve growth factor (NGF) and Ca. Amplification of TA cells is inhibited: retinoic acid and transforming growth factor beta (TGF3).

Ocular surface disorders, caused by the absence of corneal stem cells, are located only in the basal limbal epithelium. Pro arguments are represented by the investigation of cellular differentiation; human basal limbal epithelium lacking the expression of differentiation related Keratins (K3). After the simultaneous loss of the corneal and limbal epithelium, the corneal phenotype can be reconstituted by the transplantation of limbal stem cells [**4**-**6**]. The neoplasm of the corneal epithelium always originates from the limbal epithelium [**4**-**6**].

## Etiology of insufficiency of the limbal epithelium (stem cells deficiency)

Stem cells deficiency (SCD) can be primary and secondary.

The primary form is represented by the absence of external factors as injuries, mechanical damages, pharmaceutical drugs, aniridia (irregular and cloudy epithelium, corneal vascularisation) and congenital erythrokeratodermia described by Burns [**4**-**6**] (irregular and clear epithelium-traverse by blood vessels).

The secondary form is represented by chemical and thermal burns, contact lens wearer (CL related epithelial dysfunction (CLRD) [**5**]), and limbal surgery: excision of limbus for tumors, excision of pterygium, cryosurgery for ciliary body and Steven-Johnson syndrome. In case of chemical burns, the mechanism is through limbal epithelial damage, ischemia of the limbal vessels with increased permeability, influx of leucocytes in the epithelium and stroma, with alteration of the regulation of cellular proliferation and differentiation [**4**-**6**] and invasion of conjunctival epithelium.

The symptoms of stem cell deficiency are decreased vision, photophobia, tearing, blepharospasm, recurrent episodes of pain, chronic inflammation.

Seen at the slit lamp examination, the signs are represented by a dull and irregular reflex of epithelial reflex, the deep layers of the epithelium and anterior stroma containing blood vessels and area of opacification, ingrowths of thickened fibrovascular pannus and calcification (in severe absence of corneal epithelial stem cells).

The histological diagnostic tools are represented by the impression cytology, Goblet cells – containing conjunctival epithelium on the corneal surface. In advanced disease, conjunctival Goblet cells may be completely absent.

The immunohistochemical (monoclonal antibodies) diagnostic tools are: absence of cornea type differentiation (absence of keratin CK3, 12), presence of conjunctival phenotype (CK 19), presence of mucin in Goblet cells.

## Management of limbal stem cell deficiency

The patient examination consists in eye lid examination (position, movement during blinks, lid margin anatomy, trichiasis, lagophthalmos, ankyloblepharon), wetting of the ocular surface (Schirmer’s test, BUT, patency of the lacrimal puncta and canaliculi), thickness of the fibrovascular pannus (slit lamp examination/ UBM/ anterior segment OCT), IOP, estimation of the visual potential of the eye (B-scan echography, electrophysiological tests – ERG/ PEV) and a thorough workup of the patient (baseline blood tests, HLA).

The mini-scleral contact lenses can be used in stem cells deficiency. The advantages are the following: they are smaller than scleral contact lenses (15-18 mm), easier to fit and handle by the patient, provide a cushioning layer of the fluid for the cornea to rest in, useful in dry eye and reduce inflammation in patients with moderate to severe affection of the cornea (Stevens Johnson Syndrome).

## Treatment of deficiency of stem cells

The types of stem cells used for treatments are allogenic stem cells-derived from a genetically different donor within the same species [**4**], autologous mesenchymal stem cells (derived from the patient prior to use in various treatment) [**14**] and xenogeneic stem cells (derived from different species and used for research purpose) [**12**].

## Historical data about transplantation of stem cells

The first successful transplantation of corneal stem cells into damaged eyes to restore vision was performed in 2003; it used sheets of retinal cells from aborted fetuses [**12**].

In 2005, the vision of 44 eyes [**11**] in Queen Victoria Hospital Sussex England (Dr. Daye) was restored with this technique, using the adult stem cells from the patient, a relative, or even a cadaver.

In 2009, stem cells collected from human corneas, which can restore transparency without provoking a rejection response, were used in the University of Pittsburg Medical Center [**14**].

In Jan 2012, Dr. Stephen Swartz from UCLA Stem Eye Institute operated on two women who were legally blind from macular degeneration. Improvements of vision were noted after retinal injection of human embryonic stem cells [**10**].

## The algorithm for surgical intervention

No surgery is needed in case of partial stem cell deficiency (SCD) with conjunctival epithelium on the cornea and visual axis not involved. When the visual axis is involved, a sequential sector conjunctival epitheliectomy (SSCE) should be performed. Auto limbal transplantation is recommended in total unilateral SCD. Allografts from the living related donor/ cadaver donor are performed in total bilateral SCD.

## Surgical techniques

1. Sequential sector conjunctival epitheliectomy

It is performed in mild to moderate or partial SCD. It is carried out under topical anesthesia, at slit lamp. The abnormal epithelium is gently scraped or brushed off with a crescent blade (to clear the visual axis/ complete removal of all abnormal epithelium up to the limbus). The postoperative treatment consists of topical antibiotics, artificial teardrops, and bandage contact lens.

2. Auto limbal and living related donor limbal transplantation

It is used in active inflammation. It provides fresh tissue and has a very low risk of long-term donor site complications. The donor conjunctiva should not include tenon’s capsule. Amniotic membrane can be used as a graft (if the bed is scarred and unhealthy) or as a patch.

The donor tissue is harvested under topical, local, or general anesthesia.

In preparation of the recipient eye, a peribulbar subtenon’s or general anesthesia is performed, followed by a 360-degree peritomy – 1-2 mm peripheral to the visible limbus, with the removal of the fibrovascular pannus covering the cornea. It is important to find the plane of the least resistance and extend that across the whole cornea. The thin abnormal tissue is mechanically scraped off the cornea.

## Use of amniotic membrane

Amniotic membrane graft - 9-10 mm (used to become part of the recipient) is used when the exposed corneal stroma is found to be rough and irregular. An amniotic membrane patch is intended to afford cover and later is removed/ expected to fall off.

The donor explants are sutured at 12 and 6 o’clock of the recipient cornea. It is placed with the conjunctival edge posterior and the corneal edge anterior, sutured with a monofilament 10.0. Topical antibiotics, artificial teardrops, and bandage contact lens are prescribed post operatively.

3. Allo-limbal cadaver transplantation

In the acute stage of disease, explants are harvested from the cadaver. The disadvantage is that the tissue is not immunocompatible and there is a risk of rejection. The explants are taken from the whole enucleated globe or from a sclero-corneal disk.

The preparation of the recipient eye and the suturing of the donor explants are similar as in auto limbal grafts.

## References

[1] Hall PA, Watt FM (1989). Stem cells: the generation and maintenance of cellular diversity. Development.

[2] Potten CS, Morris RJ (1988). Epithelial stem cells in vivo. J Cell Sci.

[3] Gordon JI, Schmidt GH, Roth KA (1991). Studies of intestinal stem cells using normal chimeric, and transgenic mice. FASEB J.

[4] Kruse FE, Tseng SCG (1993). Growth factors modulate clonal growth and differentiation of cultured rabbit limbal and corneal epithelium. Invest Ophthalmol Vis Sci.

[5] Kruse FE, Tseng SC (1992). Proliferative and differentiative response of corneal and limbal epithelium extracellular calcium in serum-free clonal cultures. J Cell Physiol.

[6] Kruse FE, Tseng SCG (1993). A tumor promoter-resistant subpopulation of progenitor cells is larger in limbal epithelium than in corneal epithelium. Invest Ophthalmol Vis Sci.

[7] Davanger M, Evensen A (1971). Role of the pericorneal peripapillary structure in renewal of corneal epithelium. Nature.

[8] Ebato B, Friend  J, Thoft RA (1987). Comparison of central and peripheral human corneal epithelium in tissue culture. Invest Ophthalmol Vis Sci.

[9] Ebato B, Friend  J, Thoft  RA (1988). Comparison of limbal and peripheral human corneal epithelium in tissue culture. Invest Ophthalmol Vis Sci.

[10] Chen JJY, Tseng  SCG (1991). Abnormal corneal epithelial wound healing in partial-thickness removal of limbal epithelium. Invest Ophthalmol Vis Sci.

[11] Kenyon KR, Tseng  SC (1989). Limbal autograft transplantation for ocular surface disorders. Ophthalmology.

[12] Zieske JD, Bukusoglu G, Yankauckas MA (1992). Characterization of a potential marker of corneal epithelial stem cells. Invest Ophthalmol Vis Sci.

[13] Schofield R (1983). The stem cell system. Biomed Pharmacother.

[14] Schermer A, Galvin  S, Sun T-T (1986). Differentiation-related expression of a major 64K corneal keratin in vivo and in culture suggests limbal location of corneal epithelial stem cells. J Cell Biol.

[15] Cotsarelis G, Cheng  S-Z, Dong G, Sun  T-T, Lavker RM (1989). Existence of slow-cycling limbal epithelial basal cells that can be preferentially stimulated to proliferate: implications on epithelial stem cells. Cell.

[16] Zieske JD, Wasson  M (1993). Regional variation in distribution of EGF receptor in developing and adult corneal epithelium. J Cell Sci.

